# Editorial: Anthropogenic Impacts on the Microbial Ecology and Function of Aquatic Environments

**DOI:** 10.3389/fmicb.2016.01044

**Published:** 2016-07-06

**Authors:** Maurizio Labbate, Justin R. Seymour, Federico Lauro, Mark V. Brown

**Affiliations:** ^1^School of Life Sciences and the ithree Institute, University of Technology SydneySydney, NSW, Australia; ^2^Climate Change Cluster, University of Technology SydneySydney, NSW, Australia; ^3^Singapore Centre on Environmental Life Sciences Engineering, Nanyang Technological UniversitySingapore, Singapore; ^4^Asian School of the Environment, Nanyang Technological UniversitySingapore, Singapore; ^5^School of Biotechnology and Biomolecular Sciences, University of New South WalesSydney, NSW, Australia

**Keywords:** pollution and global change, climate change impacts, aquatic microbiology, resistance gene reservoirs, microbial community disturbance

Since, the beginning of the industrial revolution, anthropogenic pressures on natural environments have steadily escalated due to increased human population size, modification and destruction of habitats, and pollution. This has led to ecosystem degradation and widespread extinction of plant and animal species. In recognition of this unprecedented human impact, a new geological time period, labeled the Anthropocene, has been defined as the time period during which humans have significantly impacted the Earth's geological and ecological systems (Waters et al., 2016). During the last 40 years, a growing awareness of the negative impacts of humankind on the Earth has led to enactment of government legislation and a degree of modulation of human behavior. However, the emphasis of these changes in attitude and practice has typically been on the conservation of plants and animals, while the effect of anthropogenic activity on natural microbial populations has been largely ignored. This is a significant oversight because microbial communities are generally the first responders to environmental perturbation and can either augment or buffer environmental shifts via, often complex, positive, and negative feedback loops.

Microbes are the most abundant organisms in aquatic ecosystems playing key roles in ecosystem productivity and biogeochemistry. The impacts of anthropogenic activity on the ecology and function of aquatic microbial assemblages are multifarious and often largely undefined, but the advent of powerful new tools including next generation sequencing and novel modeling approaches has begun to shed light on this important question (Hunt and Ward; Tan et al.). The publications presented in this Research Topic are diverse with many, perhaps unsurprisingly, that address the impacts of two of the most important problems facing microbes in aquatic systems, climate change, and pollution.

One of the major impacts of climate change on marine and aquatic ecosystems is an increase in sea surface temperature (SST). In their contribution, Tout et al. demonstrated that increasing SST disrupts the *Pocillopora damicornis* coral microbiome, increasing the occurrence of *Vibrio* species and in particular the coral pathogen *V. corallilyticus.* In light of previous evidence that *V. corallilyticus* can lead to coral bleaching (Ben-Haim and Rosenberg, 2002), these patterns provide evidence for an additional mechanism in the mass bleaching events that occur during elevated SSTs. Similar observations of increasing influence of microbial pathogens due to rising SST are being made in other systems. The Petton et al. contribution demonstrated that increasing SSTs are increasing the threat to an important aquaculture species, the Pacific Oyster (*Crassostrea gigas*), which was found to be more susceptible to the ostreid herpes virus (OsHV-1) at an elevated SST. As with the Tout et al. study, disruption of the *C. gigas* microbiome and replacement with *Vibrio* species was revealed to be an important factor in the full expression of disease by OsHV-1 (Petton et al.).

In Europe, warming SST has led to increases in *Vibrio* infections in both humans and marine animals (summarized in Le Roux et al.), which catalyzed the first European workshop (Paris, 2015) aimed at forming a research network to improve understanding of the factors driving *Vibrio* disease events and their impact on human and ecosystem health and, food security (Le Roux et al.). *Vibrio* disease events and pathogen evolution are inextricably linked to how humans interact with their aquatic environment. For example, the dense human population and low-lying geography of the river deltas is hypothesized to have allowed regular intrusion of *Vibrio cholerae* into human drinking water allowing for adaptation of this deadly pathogen to the human gut (Boucher et al.). Based on the conclusions of the workshop, rising SSTs are likely to result in increased and/or more severe disease outbreaks requiring further research to develop strategies for prevention and mitigation. Continuing with the theme of climate change, a potential partial solution to this problem is CO_2_ capture and storage in sub-seabed reservoirs however, Rastelli et al. sounded a warning that CO_2_ leakage from such reservoirs could significantly impact benthic microbial communities affecting carbon cycling and nutrient regeneration processes.

In addition to the effects of climate change, anthropogenically-derived water pollution is another major impact within aquatic ecosystems. Contributions to this special issue indicate that influx of pollutants and nutrients into aquatic systems significantly disrupts the structure and function of natural microbial assemblages, leading to reduced species diversity, increased heterotrophy and rises in the numbers of potentially virulent/toxic microbes (Moreira et al.; Quero et al.; Jeffries et al.). Some of these localized pollution events can also amplify the effects of global-scale climate change, by further stimulating events such as the *Vibrio* blooms described above or cyanobacterial or algal blooms that lead to mortality of aquatic animals through anoxia or production of toxins (Rastogi et al.).

Beyond nutrient pollution, antibiotics and antibiotic-resistance genes are emerging as significant pollutants in aquatic ecosystems (Zhang et al.). The worsening antimicrobial resistance crisis is now being acknowledged as a One Health issue where under this concept, the health of humans is recognized as being inextricably linked to animal and environmental health. In an environmental context, aquatic environments are a major reservoir for resistant microbes and resistance genes as a result of excessive use of antimicrobials and untreated effluent streams (Michael et al., 2014). Sub-inhibitory concentrations of antibiotics in aquatic environments promote antibiotic resistance in bacteria and significant changes in DNA sequence (e.g., mutation) and genome architecture such as inversions and deletions in the bacterial chromosome (Chow et al.) and, affect biogeochemical cycles (Balcazar et al.). Furthermore, natural biofilms are reservoirs for resistance genes with the close proximity of bacteria to one another promoting lateral gene transfer processes (Balcazar et al.). More research is required to elucidate the role of antibiotic waste on ecological systems and what impact these resistance reservoirs have in promoting antibiotic resistance in clinical environments.

A key point raised by Jeffries et al. is that we lack benchmark data for many of our urbanized estuaries, limiting our ability to assess the impact of anthropogenic activity on these important and valued ecosystems. We also do not know whether a microbial community can be restored following a disturbance (chronic or acute) and what impact industrial pollutants have on the biogeochemistry of an environment (Quero et al.). Although not a result of pollution, these questions are answered in part by Bernhard et al. who reported that disturbance of a natural community due to tidal restrictions had long-lasting impact on sediment nitrogen-cycling bacterial communities even 30 years after restoration. Tan et al. also addressed the question of how artificial urban structures affect microbial assemblages. Regarding restoration, Aracic et al. reviewed numerous currently available biological approaches that could be applied. However, new methods are required to understand how microbial communities shift when disturbed to better understand the resilience of microbial communities and to assist in identifying the best ways of restoring them. Within this context, Tan et al. examined the utility of next generation sequencing approaches for assessing water quality, while Hunt and Ward presented a promising network modeling approach for predicting how disturbances transmit through microbial interactions. Both of these studies provide crucial direction for future studies in determining how anthropogenic processes disturb microbial communities and how (if possible) do we restore them.

Our aquatic ecosystems are highly valued as sources of food (Lu et al.) and recreation (Jeffries et al.) however, they are experiencing unprecedented levels of impacts from human activities particularly those of pollution and climate change processes. The diverse contributions to this Research Topic have highlighted the extent to which natural communities of aquatic microorganisms are faced with environmental perturbation and degradation as a consequence of human activities. With more research and better understanding combined with better public awareness and government policy (Rastogi et al.), improved management of, and restoration of these environments can proceed. The impetus for such change is not easily found, however, aquatic environments are often, and increasingly, highly valued by the human populations living near to them. With effective public communication, this can be used to drive change, as is happening in the 2016 Olympic city of Rio de Janeiro with its massive restoration of Guanabara Bay (Fistarol et al.).

We thank all of the participating authors for their contributions to this issue, which we believe will be a valuable resource as aquatic ecosystems continue to experience increasing anthropogenic impacts into the future.

## Author contributions

ML, JS, FL, and MB contributed to the writing of this editorial and approved its content.

## Funding

MB was supported by an Australian Research Council (ARC) grant DP150102326. JS was supported by an ARC Future Fellowship FT130100218.

### Conflict of interest statement

The authors declare that the research was conducted in the absence of any commercial or financial relationships that could be construed as a potential conflict of interest.

## References

[B1] Ben-Haim Y. Rosenberg E. (2002). A novel *Vibrio* sp. pathogen of the coral *Pocillopora damincornis*. Mar. Biol. 141, 47–55. 10.1007/s00227-002-0797-6

[B2] Michael C. A. Dominey-Howes D. Labbate M. (2014). The antimicrobial resistance crisis: causes, consequences and management. Front. Public Health 2:145. 10.3389/fpubh.2014.00145 25279369 PMC4165128

[B3] Waters C. N. Zalasiewicz J. Summerhayes C. Barnosky A. D. Poirier C. Gałuszka A.. (2016). The anthropocene is functionally and stratigraphically distinct from the holocene. Science 351:aad2622. 10.1126/science.aad2622 26744408

